# Gamification in health education: a mini-review of engagement strategies for the smartphone generation

**DOI:** 10.3389/fpubh.2026.1816727

**Published:** 2026-04-08

**Authors:** John Matkovic

**Affiliations:** Department of Health Sciences, Illinois State University, Normal, IL, United States

**Keywords:** academic success, gamification, health education, smartphone, student engagement

## Abstract

Smartphones and social media use among young adults is at an all-time high, and research has linked non-academic use to reduced long-term retention and exam performance. Students often perceive device use as a social device with little to no risk to their academic success, while social norms research documents frequent misperceptions of peer smartphone behaviors. Across health professions and health sciences education, gamification (e.g., interactive quiz platforms, gamified web modules) shows potential to improve knowledge and motivation. This mini-review examines research on the use of smartphones among college students, the effects of smartphone distractions in college classrooms, the limitations of traditional lecture-based instruction in modern learning environments, and how gamification can support applied learning in health education/public health. Lastly, this mini review suggests taking advantage of the opportunity that smartphones present in the classroom and offers recommendations regarding gamification for educators and researchers in health education and public health.

## Introduction

Smartphone use and smartphone distractions may now be permanent features of the college learning environment. Studies indicate that students commonly bring phones to class and engage in non-class-related activities (e.g., texting, social media) during instruction. In a (year) survey of college students, 95% reported bringing their phones to class daily, and 92% reported texting during class (1). In a survey of U.S. college students, respondents reported spending more than one-fourth of class time using a digital device for non-class purposes on class days (2). Additionally, 63% of young adults aged 18–29 report using the internet constantly (3). Beyond just universal uptake, findings show that smartphone addiction has also risen to worrying levels (4). This use implies a decrease in attention to class material.

## Smartphones, digital distractions, and academic outcomes

While the focus on smartphones in the classroom is often negative, there is also evidence that smartphones are powerful tools that can be used in a variety of ways for academic success. Students can read textbooks, complete homework, and access online course content through their smartphones, making learning more mobile and accessible than ever before. This is complicated, however, by distractions that may arise when smartphones are used as learning tools. In a review by Martin et al. (5), over half of all academic distractions attributed to technology impacting personal reading, comprehension, and academic multi-tasking. To further emphasize this point, students who report non-academic multitasking (browsing social media, using the internet, texting) have more difficulty recalling content (6), perform poorer academically the more frequent the use (7), and can be distracted just from having a phone nearby (8). These effects may be amplified if academic work is being completed on or with the use of smartphones, as distractions and notifications may be immediately apparent.

It is important to identify factors that may negatively influence college success because engagement and academic performance are tied to persistence and dropout intentions, as explained by Xie and Ke (9) and Trolian and Jach (10). Numerous studies have shown a link between self-reported in-class phone use and test performance, finding that greater in-class phone use was significantly and negatively associated with test scores (11). Additionally, consistent associations have been found between smartphone use and lower Grade Point Average (GPA) (12). Importantly, Gingerich and Linewaver (13) demonstrated that phone use (specifically texting) can negatively impact grades, suggesting that links between phone use and academic performance might not be solely correlational. Glass and Kang’s (12) findings also suggest that in-class distractions from electronic devices can negatively impact student retention. The body of literature on phones and electronics indicates that these devices can cause distractions, and that distracted students face additional challenges in paying attention, recalling content, and succeeding academically.

These distractions are particularly problematic for health education and public health due to the applied nature of the field. From practical epidemiology (14) to monitoring food, water, and planning for emergency preparedness (15), the applications of public health are everywhere. It is evident that Public Health and Health Education, like many areas of focus, require attention, professional practice, and applied methodology, which can be challenging in low-engagement environments.

## Digital devices, smartphones, and social norms

In academic settings, Briskin et al. (16) found that positive social norms may increase the likelihood that others find these behaviors acceptable within student groups, thereby increasing overall phone use. Smartphone-use social norms are strongly influential on classroom usage behaviors. Social norms theory holds that people can be influenced through both descriptive norms (beliefs about what others do) and injunctive norms (beliefs about what others approve of) (17, 18). In an extensive review, Fuchs (19) found that social norms play a significant role in predicting phone-use behaviors, in that participants’ subjective norms strongly affected their intention to use their phone, and Xu et al. (20) further showed that peer pressure also significantly contributed to social media phone use. Beyond this, several studies indicate students often overestimate peers’ smartphone app use (21, 22), suggesting a mismatch between perception and actual behavior that may be driving increased use.

## Traditional and experiential learning public health education

If smartphone-driven distraction reduces retention and academic outcomes and may increase the likelihood of leaving college without completing a degree, then alternative approaches to teaching that consider and utilize smartphones should be explored. In the US, traditional lecture-based learning has long been the standard. However, as students and their behaviors change, it may be necessary to adjust the classroom educational approach. Studies have shown that “active” or “experiential” learning, as opposed to traditional lecturing, may be more effective for student learning (23). Further, the Council on Education for Public Health (CEPH) (24), the independent accrediting agency for public health programs in the US, recommends that student competency should be measured and assessed through applied and practical experiences. This is further supported by Pham et al. (25), who show that experiential learning is important for engaging future public health professionals.

## Gamification as a strategy for health education

The term “gamification” has been used loosely to describe the use of various elements of gaming and game design for purposes not solely related to recreational gaming, fun, or play. The first accepted use of the term “gamification” emerged in the early 2000s but did not become more widespread until years later (26, 27). The definition that this mini-review will use is “the use of game design elements in non-game contexts” as developed by Deterding et al. (28).

According to a sweeping review by Krath et al. (29) gamification address interest and engagement in education by providing clear and relevant goals, allowing individual goal-setting, with immediate feedback and positive reinforcement. It also supports engagement by allowing comparison with peers, addressing social norms through gamification use, using content that can be adjusted for each person’s experience, allowing for guided paths, with multiple options for completing work, and lastly, by simplifying the learning experience. It is suggested that games and gamification enhance learning by improving engagement, motivation, and participation in work or educational content (29, 30).

In health curriculum, several studies suggest meaningful gains in knowledge and engagement when gamification is used to deliver course content. For example, Kahoot! is a learning platform that can be accessed online or via an app, where teachers and instructors can develop and deliver quizzes, polls, games, and tests (31). Cortes-Perez et al. (32) found that using Kahoot! to deliver exam content helped students study and correctly answer more test questions than students who did not receive exam content via Kahoot! In another example, Kahoot! was successfully used to improve vaccine knowledge among nursing students when Kahoot! educational games were used, compared to students who participated in standard nursing student curriculum (33). Aloum et al. (34) also demonstrated that utilizing web-based games specifically designed for medical students significantly increased test scores and understanding of relevant course concepts. Finally, Patil et al. (35) demonstrated that a pilot activity utilizing AI to deliver public health content aligning with CEPH competencies was enjoyable and engaging to students. Based on the demonstrated increases in knowledge and acceptability seen in students, then, gamification may help to address disengagement related to smartphone use seen in the classroom.

## Mechanisms for gamification in learning

In educational settings, gamification typically includes specific game elements such as points, positive feedback loops, competition, time-bounded challenges, or reward mechanics embedded in existing coursework (e.g., quiz platforms such as Kahoot!) (31–33). These gamification elements are thought to influence students by increasing motivation and attention, making participation more relevant, providing immediate feedback, and making goals and progress clearer (29, 30). Krath et al. (29) highlight that gamification research often focuses on motivational and behavioral explanations, but theory use is not always explicitly stated, underscoring the need to identify and describe theoretical pathways in future empirical research.

For a possible gamification framework, engagement is the first step of the pathway to attention and academic performance. Engagement, however, is not a single construct. As Fredericks et al. (36) explain, engagement can be behavioral (classroom participation), cognitive (a person’s willingness to be challenged), and affective (a person’s level of interest, enjoyment, or the sense of belonging one feels in class) (9, 10). Gamification can be viewed as a design strategy intended to increase engagement behaviors known to predict learning and educational persistence. This may help explain why gamified interventions have been shown to improve learning outcomes, including knowledge, satisfaction, interest, and classroom collaboration (32, 33). There may also be a broader reason gamification finds success. Evidence indicates active approaches are more successful than passive learning, gamification might succeed as a method to support and improve active learning, rather than replace it (23). Further, the engagement–academic-success pathway has been supported by the literature in higher education: active student engagement predicts academic performance and persistence in online learning environments, and student-faculty interaction/engagement is associated with persistence outcomes (9, 10). The mechanisms involved in gamification can directly address student attention and digital distraction in modern learning environments.

As indicated earlier in this mini-review, in-class digital device multitasking is commonplace and can impair learning outcomes, with experimental and quasi-experimental evidence indicating that devices can reduce cognitive capacity and overall academic performance (6, 8, 11). In this context, gamification may work by redirecting students’ attention, encouraging them to use their devices for course participation rather than off-task distractions (e.g., quiz participation, assignment feedback, team tasks) (31–33). This is consistent with literature showing that students’ behavioral intentions to use smartphones in class are shaped by perceived usefulness, with social norms theory indicating that perceived norms can shape behavior (17–19, 37). Gamification elements involving group or shared participation (e.g., group assignments, visible and shared progress) may create positive descriptive norms around engaging with course content and may help to limit the harmful side effects of off-task device use. These components suggest a possible pathway from gamification to learning outcomes: gamification elements can lead to increased engagement and perceived relevance, which can lead to improved learning outcomes.

## Theory in gamification

Although there has not been a single consistent theoretical approach to gamification in the literature, Krath et al. (29) show that gamification research has drawn heavily on self-determination theory, flow theory, and experiential learning theory. This may signal that using gamification strengthens students’ feeling of competency and autonomy, supports immersive learning, and helps connect coursework to real-world practice. Literature also suggests that how gamification is used is also important to its success. Intentionally selecting game elements (e.g., mastery-based feedback; including meaningful choices for students; or providing scenarios for experiential learning) is important because it can help prevent “pointification.” Pointification can occur when participation is temporarily increased because of something new or novel, but this same participation does not result in lasting learning outcomes (29).

The literature also suggests that gamification’s impact on attention and behavior is likely affected by social influence processes, which have implications for applying gamification in the classroom. Norms theory states that students’ behaviors are affected by perceptions of what their peers do and what their peers approve, particularly when the perceptions are highly relevant, like observing class participation by peers (17, 18). Students often misperceive peer digital behavior and may overestimate how much others engage with social media or smartphone use, which can normalize off-task device use and increase pressure to remain constantly connected (21, 22). Further, peer pressure and normative beliefs are linked to problematic or habitual smartphone behaviors, implying that use of digital devices in class becomes reinforced by social cues (20, 16). This means gamification may be both motivating and norm-shaping: visible shared participation can help reset descriptive norms of positive classroom engagement.

Gamification outcomes often appear most impactful when working within technology acceptance and active learning frameworks, because these theories help explain why device-mediated learning succeeds. Technology adoption in educational contexts is commonly explained using the Technology Acceptance Model (TAM). The TAM explains that perceived usefulness and perceived ease of use shape behavioral intentions to adopt technology (37). These constructs are particularly relevant for gamified learning environments, because they strongly influence students’ willingness to engage with digital tools in the classroom (19, 27). Evidence suggests gamified learning tools may raise perceived usefulness by making participation immediate, more rewarding, and more social, which is supported by health professions education studies using Kahoot! and other gamified methods that emphasize regular recall and feedback (32–34).

## Smartphone restrictions and learning outcomes

Despite the growing literature that suggests gamification can have positive academic benefits, evidence also points to banning devices as a means of improving learning. Students who multitask more perform poorer in classes than others, and students have performed better in their classes when devices are banned (9, 23). Evidence also has linked device bans in schools to better scores on standardized tests (38). In fact, 41 states in the United States have now adopted policies or laws related to device usage in K-12 classrooms (39). While this is a consideration in the implementation of gamification in the classroom, it may be true that gamification can present a directed purpose for occasional device use. In addition, gamification need not require personal device use. Using gamification through more traditional methods (e.g., classroom competitions), may help students who are used to the constant presence of a device to engage and remain actively attentive in the classroom.

## Discussion

The current state of education and Pew Research Center (3) data suggest one important thing: smartphone use among students is unlikely to decrease anytime soon. The literature has consistently shown that digital devices can have negative effects on grades, academic success, and retention. Evidence indicates that reducing phone access during class can improve comprehension, recall, and test scores.

However, in higher education, smartphones can also be used as tools: to answer questions live in class, respond to polls, and research and find answers during course discussions. Given the universal adoption of phones as not just social devices but also essential tools for students, it is vital that new approaches be explored to leverage these already-present devices to benefit students.

Thus, this mini-review highlights several important points. First, smartphones and digital devices can be viewed not only as a challenge to learning, but as an opportunity. With the rise of web-based games and apps like Kahoot!, instructors can leverage powerful tools that almost all students already have. Second, at a minimum, the use of gamification in health professions education indicates increases in both engagement and attention (32, 33). With the rise of digital distractions, improving engagement and attention may yield significant benefits in the long term for students and health professionals alike. As CEPH (27) and Pham et al. (28) describe, experiential learning is vital to health professions. Improving instructors’ ability to provide novel, experiential activities can be challenging, and approaches to enhance experiential learning are worth exploring. Instructors in health education should explore using gamification (specifically those with immediate feedback and experiential scenarios) as a means of enhancing student engagement, and student interest in courses where applied learning is vital.

Despite the opportunities that gamification offers in education for health professionals, several limitations must be acknowledged. As outlined in this mini-review, “gamification” and its use are still relatively new. Given that gamification as a practice is still emerging, the body of literature connecting it to health professions and education outcomes is continuing to grow. The literature examined in this mini-review is also limited, as studies on gamification and health education have not been longitudinal. While the current literature shows promising results and indicates “gaming” elements have positive benefits, the long-term effects on learning are still not fully known. In the context of health professions education and gamification, literature is limited. Few studies have explicitly examined the use of gamification to improve the education of CEPH competencies, needs assessment, program planning and evaluation, health coaching, or health communication, or Master of Public Health (MPH) curricula. It must also be recognized that, in the spirit of equity, gamified learning may not always be suitable, especially for students without access to smartphones or other digital devices. Additionally, instructors should be cautious with purely competitive game elements, as too much competition could disengage some learners. Finally, there is a growing push to ban digital devices in classrooms, and evidence does indicate such restrictions help learning. Despite these limitations, the benefits of gamification in learning are promising. Importantly, gamification is not dependent on digital devices. Instructors can utilize in-person gamification, the use of non-phone-based clickers for quizzes, or simply physical games like board games or role-playing activities.

Gamification of health education is an opportunity to deliver impactful experiential learning across a variety of health professions. Many MPH programs in the United States are fully online; including games and game elements in the curriculum could add an interactive element to this space. Further, in a time and environment where funding for real-world experiences is shrinking, the use of gamification may help fill the gap left by budget shortfalls. With the advent of web-based platforms, smartphones, and AI, utilizing these tools for gamification presents a novel learning opportunity with a relatively low barrier to entry for students. Future work should examine the use of gamification in MPH courses to identify whether these same benefits emerge in online MPH learning spaces.

Health education would benefit from research conducted to identify whether gamification can improve experiential learning related to core public health competencies. Longitudinal studies are also needed to examine the long-term effects on educational outcomes and determine whether the benefits of gamification persist beyond a single intervention or course. With smartphones being in the hands of almost every student, and device restrictions being fragmented and inconsistent, efforts should be made to turn these devices from distractions into delivery systems for engaging, quality course content.
